# Predicting the Survival Probability of Neuroendocrine Tumor Populations: Developing and Evaluating a New Predictive Nomogram

**DOI:** 10.1155/2021/9126351

**Published:** 2021-01-28

**Authors:** Jian-Xian Chen, Yan Lin, Yi-Liang Meng, Ai-Xia Zhao, Xiao-Juan Huang, Rong Liang, Yong-Qiang Li, Zhi-Hui Liu

**Affiliations:** ^1^Department of Medical Oncology, Guangxi Medical University Cancer Hospital, Nanning 530021, China; ^2^Department of Oncology, Baise People's Hospital, Baise 533000, China

## Abstract

**Purpose:**

The purpose of this study was to develop and initially validate a nomogram model in order to predict the 3-year and 5-year survival rates of neuroendocrine tumor patients.

**Methods:**

Accordingly, 348 neuroendocrine tumor patients were enrolled as study objects, of which 244 (70%) patients were included in the training set to establish the nomogram model, while 104 (30%) patients were included in the validation set to verify the robustness of the model. First, the variables related to the survival rate were determined by univariable analysis. In addition, variables that were sufficiently significant were selected for constructing the nomogram model. Furthermore, the concordance index (C-index), receiver operating characteristic (ROC), and calibration curve analysis were used to evaluate the performance of the proposed nomogram model. The survival analysis was then used to evaluate the return to survival probability as well as the indicators of constructing the nomogram model.

**Results:**

According to the multivariable analysis, lymphatic metastasis, international normalized ratio (INR), prothrombin time (PT), tumor differentiation, and the number of tumor metastases were found to be independent predictors of survival rate. Moreover, the C-index results demonstrated that the model was robust in both the training set (0.891) and validation set (0.804). In addition, the ROC results further verified the robustness of the model either in the training set (AUC = 0.823) or training set (AUC = 0.768). Furthermore, the calibration curve results showed that the model can be used to predict the 3-year and 5-year survival probability of neuroendocrine tumor patients. Meaningfully, five variables were found: lymphatic metastasis (*p* = 0.0095), international standardized ratio (*p* = 0.024), prothrombin time (*p* = 0.0036), tumor differentiation (*p* = 0.0026), and the number of tumor metastases (*p* = 0.00096), which were all significantly related to the 3-year and 5-year survival probability of neuroendocrine tumor patients.

**Conclusion:**

In summary, a nomogram model was constructed in this study based on five variables (lymphatic metastasis, international normalized ratio (INR), prothrombin time (PT), tumor differentiation, and number of tumor metastases), which was shown to predict the survival probability of patients with neuroendocrine tumors. Additionally, the proposed nomogram exhibited good ability in predicting survival probability, which may be easily adopted for clinical use.

## 1. Introduction

The endocrine system is composed of neuroendocrine cells that exist throughout the body. These cells can be found in isolation or form small aggregates from which neuroendocrine tumors arise [1]. From 1973 to 2004, the incidence of neuroendocrine tumors (NETs) reported in the United States increased significantly from 1.09/10 million to 5.25/10 million [2]. One of the important clinical features of neuroendocrine tumors is heterogeneity. Neuroendocrine tumors can be invasive or inert, and they may be asymptomatic or have symptoms that are completely different. This is because these tumors secrete different neuropeptides or biogenic amines. Additionally, neuroendocrine tumors may be nonfunctional or functional [3]. Due to their complex and heterogeneous biological behavior, the prognosis of patients with NETs remains unclear. Therefore, it is important to understand the biological and clinical characteristics of NETs and how they affect the prognosis of specific individuals.

Currently, the most common prediction systems for NETs are the American Joint Committee on Cancer (AJCC) and European Society of Neuroendocrine Tumors (ENETS) TNM staging system, which are based on the depth of tumor infiltration (T), nodules of metastatic lymph nodes (N), and distant metastasis (M) [1]. In addition, the World Health Organization (WHO) classification standard proposed in 2010 is commonly used, which divides neuroendocrine tumors into neuroendocrine tumors G1 (NET G1), neuroendocrine tumors G2 (NET G2), and neuroendocrine tumors G3 (NEC G3), as well as mixed adenocarcinoma and neuroendocrine carcinoma (MANEC). The main parameters used (Ki67 proliferation index and mitotic count) provide a possible prognostic treatment for this particular type of malignant tumor [4]. However, some limitations are present in these staging systems in predicting the prognosis of patients with NETs. For example, based on the ENETS staging system, the prognosis of patients with stage I is similar to that of patients with stage IIA [5]. In addition, some studies have shown that clinicopathological characteristics may have an impact on the prognosis of patients, such as gender, age, tumor location, and primary tumor diameter [6]. Therefore, it is important to determine other variables that predict the prognosis of NET patients and build a more robust prediction model.

Among the available decision-making tools, the nomogram is currently the best and most accurate method in predicting the prognosis of cancer patients. According to its statistical definition, the nomogram is an algorithm or graphical calculation encompassing the use of multiple continuous variables in order to predict specific endpoints, such as logistic or COX proportional hazard (PHs) regression models. The impact of these continuous variables on specific results is represented on an axis, and risk points are determined according to the prognostic importance of the variables of interest [7].

Previous studies have successfully quantified the risk of certain malignancy tumors by combining prognostic factors. In addition, various studies have shown that the nomogram can predict the prognosis of patients with gastrointestinal NETs [8] and pancreatic NETs [9]. However, to the best of our knowledge, nomograms have yet to be used to predict the prognosis of neuroendocrine patients. Accordingly, this study attempts to develop and validate a nomogram model in predicting the survival probability of NET patients.

## 2. Materials and Methods

### 2.1. Patient and Tumor characteristics

In this study, 348 neuroendocrine tumor patients who were admitted to the Cancer Hospital of Guangxi Medical University, from January 1, 2013 to November 12, 2019, were enrolled. All patients were proven to have NETs, according to the surgical specimen results, histological differentiation, or immunohistochemical staining of the biopsy. This study was approved by the Ethics Committee of Cancer Hospital of Guangxi Medical University, and all patients' written consent was obtained.

The data was collected retrospectively from the patients' electronic or physical medical records.

### 2.2. Postoperative Follow-Up

All patients were followed up in outpatient clinics or by phone until December 31, 2019. The interval of follow-up was three months in the first two years after treatment and six months from 2 to 5 years. The overall survival (OS) rate was defined as the time from the start of treatment to death or the last exposure for any reason. This index was often considered to be the best end point of clinical efficacy in cancer clinical trials. If there was a small increase in survival time, it can be regarded as meaningful evidence of clinical benefit.

### 2.3. Data Preprocessing and Statistical Features of Clinicopathology

**Figure 1:**
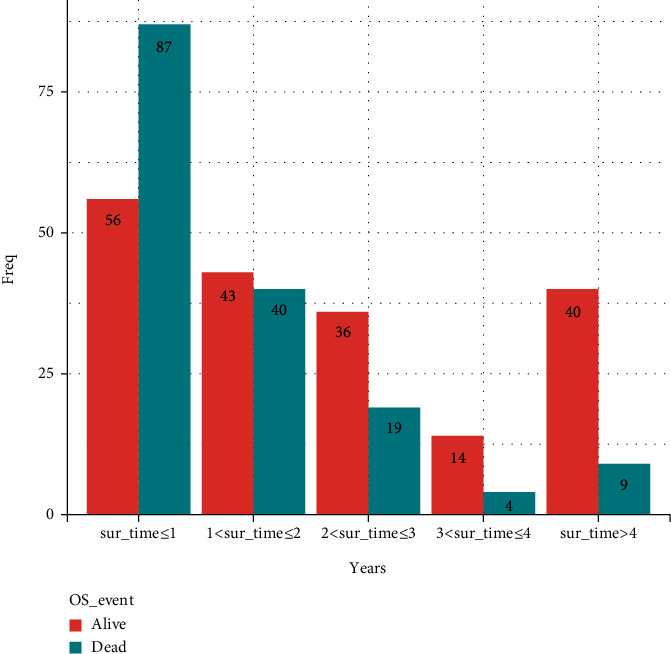
Schematic drawing of isometric sampling.

The *mice* function in the *mice* package (https://cran.r-project.org/package=mice) was used to assume that all variables were related in this study. In addition, multiple regression models were used for multiple interpolation to supplement missing values. A univariable analysis was performed using the *coxph* function in the *survival* package (https://cran.r-project.org/package=survival), and the *survminer* package (https://cran.r-project.org/package=survminer) was adopted to obtained the variables related to OS. Furthermore, the *forestplot* function in forestplot package was used to draw the forest plot. As shown in Figure 1, equidistant sampling was used. In regard to the data of live patients, 8 patients were sampled each year, and less than 8 patients were used. For that of dead patients, less than 8 patients were present for more than 4 years. Accordingly, 12 samples were extracted in the first year, while 8 samples were taken in other years.

### 2.4. Selection of Effective Feature Indicators

The *createDataPartition* function in the *caret* package was used (https://cran.r-project.org/package=caret) to divide the training set and test set according to the survival state, of which the ratio was 7 : 3. There were 131 alive and 113 dead samples in the training set, while there were 58 alive and 46 dead samples in the test set. The variables with *p* < 0.05 were selected for multivariable analysis using the *coxph* function in the *survival* package and *survminer* package in *R*.

### 2.5. Validating the Performance of the Nomogram Model

The prediction ability of the nomogram model was assessed both internally (training set) and externally (validation set) using three methods: the operator characteristic curve (ROC) analysis, concordance index (C-index), and calibration plot. C-index was an index of probability of the concordance between predicted and actual situations, ranging from 0.5 to 1.0. A calibration plot was also made, which was a graph consisting of two curves, a 45-degree straight line (perfect match) and an irregular curve (calibration curve). The operator characteristic curve (ROC) plot with area under curve (AUC) was then derived to assess the actual predictive significance of the nomogram model based on the *pROC* package [10]. C-index was obtained using the *concordance.index* function in the *survcomp* package [11]. Finally, the calibration plot was obtained via the calibrate function in the *rms* pakcage (https://cran.r-project.org/package=rms).

### 2.6. Survival Analysis

The survival analysis was performed using the Kaplan-Meier method so as to build the survival probability curves. The analysis was carried out through the functions “*survfit*” and “*ggsurvplot*” from the *R* packages “*survival*” (CRAN.R-project.org/package=survival) and “*survminer*” (CRAN.R-project.org/package=survminer to link to this page), respectively (ver. 2.42-3, available online: https://cran.r-project.org/web/packages/survival/index.html and ver. 0.4.2, available online: https://cran.r-project.org/web/packages/survminer/index.html).

### 2.7. Statistical Analysis

All data were analyzed using the *R* software and were expressed as mean ± standard deviation. Student's *t*-test was used to compare the mean between the two groups, and *p* < 0.05 was considered to be statistically significant.

## 3. Results

### 3.1. Baseline Characteristics of 348 Patients with Neuroendocrine Tumors (NETs)

As shown in Table 1, the baseline characteristics of 348 patients are listed. Here, 196 men and 152 women were present, most of whom were over 56 years old (*n* = 271, 77.87%). According to the tumor grade, 68 patients were G1 or G2 (19.54%), while 280 patients were G3 (80.46%). Moreover, most patients had lymphatic metastasis (*n* = 240, 71.26%), with no nerve invasion (*n* = 308, 88.51%). For the four indicators of hepatitis B, most patients did not have hepatitis B virus infection (negative HBsAg: *n* = 284, 81.61%; negative HBeAg: *n* = 347, 99.71%; negative HBeAb: *n* = 337, 96.84%; HBcAb negative: *n* = 331, 95.11%). Most patients had poor tumor differentiation (*n* = 282, 81.03%), and 66 patients had highly differentiated tumors (18.97%). In most patients, synaptophysin (*n* = 285, 81.90%), chromogranin A (*n* = 300, 86.21%), and nerve cell adhesion molecules (*n* = 282, 81.03%) were all negative or weakly positive. Notably, no difference was present in the clinicopathological variables between the training set and test set.

### 3.2. Variables Related to Overall Survival

**Figure 2:**
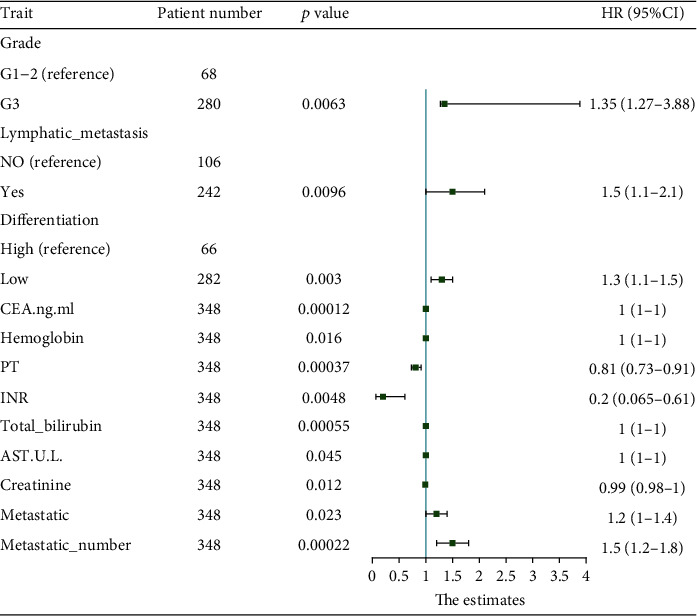
Random forest plot for single factor analysis.

**Figure 3:**
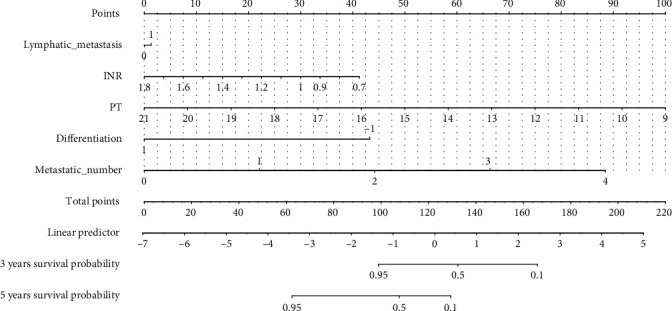
Nomogram of all survival rate for patients of NETs. NETs: neuroendocrine tumors.

When evaluating 244 patients in the training set, a univariate analysis correlated the overall survival rate with multiple factors (Table 2, Figure 2). Specifically, the 3-year survival rate of patients with tumor grade G1-2 was 37.08% (95% CI, 26.82%-51.2%), and the 5-year survival rate was 5.06% (95% CI, 1.68%-15.2%). The 3-year survival rate of patients with tumor grade G3 was 44.1% (95% CI, 36.7%-53.1%), and the 5-year survival rate was 27.8% (95% CI, 20.6%-37.6%) (*p* = 0.0063). The 3-year survival rate of patients without lymphatic metastasis was 48.4% (95% CI, 38.8%-60.5%), and the 5-year survival rate was 25.1% (95% CI, 17%-37%). The 3-year survival rate of patients with lymphatic metastasis was 38% (95% CI, 30.11%-47.9%), and the 5-year survival rate was 14.3% (95% CI, 8.33%-24.7%). The 3-year survival rate of poorly differentiated patients was 3.94% (95% CI, 24.783%-49.3%), and the 5-year survival rate was 3.49% (95% Cl: 0.898%-13.7%). The 3-year survival rate of highly differentiated patients was 45.2% (95% CI, 37.8%-54.1%), and the 5-year survival rate was 28.1% (95% CI, 20.9%-37.9%). The 3-year survival rate of patients with normal levels of carcinoembryonic antigen was 38.4% (95% CI, 29.3%-50.4%), and the 5-year survival rate was 20.1% (95% CI, 12.3%-33%). The annual survival rate was 44.5% (95% CI, 36.4%-54.5%), and the 5-year survival rate was 19% (95% CI, 12.6%-28.5%). The 3-year survival rate of male patients with normal hemoglobin levels was 25.24% (95% CI, 16.5%-38.6), and the 5-year survival rate was 8.41% (95% CI, 3.4%-20.8%). The 3-year survival rate of male patients with abnormal hemoglobin levels was 62.4% (95% CI, 48.6%-80%). The 3-year survival rate for female patients with normal hemoglobin level was 39.7% (95% CI, 28.7%-54.8%), and the 5-year survival rate was 16.7 (95% CI, 9%-31%). The 3-year survival rate for women with abnormal hemoglobin levels was 56% (95% CI, 43.3%-72.5%), and the 5-year survival rate was 24.7% (95% CI, 13.5%-45.3%). The 3-year survival rate of male patients with an abnormal level of creatinine was 40.3% (95% CI, 23.8%-68.4%), and the 5-year survival rate was 26.95% (95% CI, 10.3%-70.1%). The 3-year survival rate of female patients with abnormal level of creatinine was 53.4% (95% CI, 34.9%-81.8%), and the 5-year survival rate was 33.9% (95% CI, 17.3%-66.6%). The 3-year survival rate of patients with a normal level of PT was 47.5% (95% CI, 36.9%-61.1%), and the 5-year survival rate was 33% (95% CI, 22.8%-47.8%). The 3-year survival rate of abnormal PT was 38.9% (95% CI, 31.45%-48%), and the 5-year survival rate was 12.4% (95% CI, 7.48%-20.7%). The 3-year survival rate of patients with normal INR was 40.8% (95% CI, 34.2%-48.7%), and the 5-year survival rate was 18.9% (95% CI, 13.5%-26.4%). The 3-year survival rate of patients with abnormal INR was 49.4% (95% CI, 32.61%-74.9%), and the 5-year survival rate was 21.6% (95% CI, 8.46%-55.3%). The 3-year survival rate of patients with a normal level of total bilirubin was 42% (95% CI, 35.4%-50%), and the 5-year survival rate was 19.3% (95% CI, 13.8%-27%). The 3-year survival rate of patients with an abnormal level of total bilirubin was 38.9% (95% CI, 23.79%-63.7%), and the 5-year survival rate was 19.5% (95% CI, 8.32%-45.6%). The 3-year survival rate of patients with normal levels of AST was 41% (95% CI, 34.2%-49.1%), and the 5-year survival rate was 17.8% (95% CI, 12.5%-25.5%). The 3-year survival rate of patients with abnormal level of AST was 45.4% (95% CI, 31.2%-66%), and the 5-year survival rate was 26.5% (95% CI, 13.7%-51.1%). The 3-year survival rate of patients with tumor metastasis was 40.98% (95% Cl, 36.8%-48.2%), and the 5-year survival rate was 38.25%(95% Cl, 32.2%-45.5%). The 3-year survival rate of patients with< 1 tumor metastasis was 43.4% (95% Cl, 36.9%-51.1%), while the 5-year survival rate was 20.3% (95% Cl, 14.8%-27.8%). In the multivariate analysis, lymphatic metastasis, international standardized ratio, prothrombin time, tumor differentiation, and the number of tumor metastatic sites were found to be independent predictors of overall survival (Tables 3 and 4). Subsequently, beta coefficients from multivariate models were used to develop the nomogram (Figure 3).

### 3.3. Nomogram Predictive Ability and External Verification

**Figure 4:**
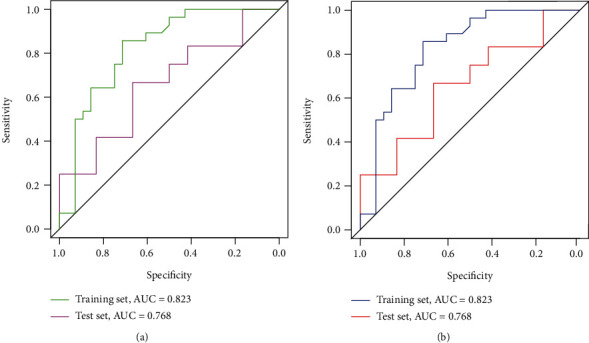
ROC curve analysis of nomogram predicting (a) the 3-year and (b) 5-year survival rate of patients with NETs. NETs: neuroendocrine tumors.

**Figure 5:**
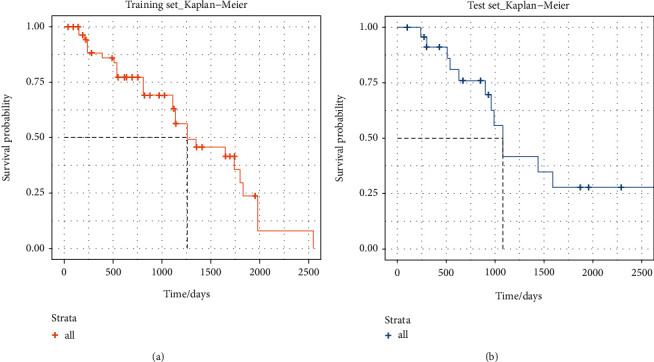
Cumulative survival of patients in the training set and test set.

**Figure 6:**
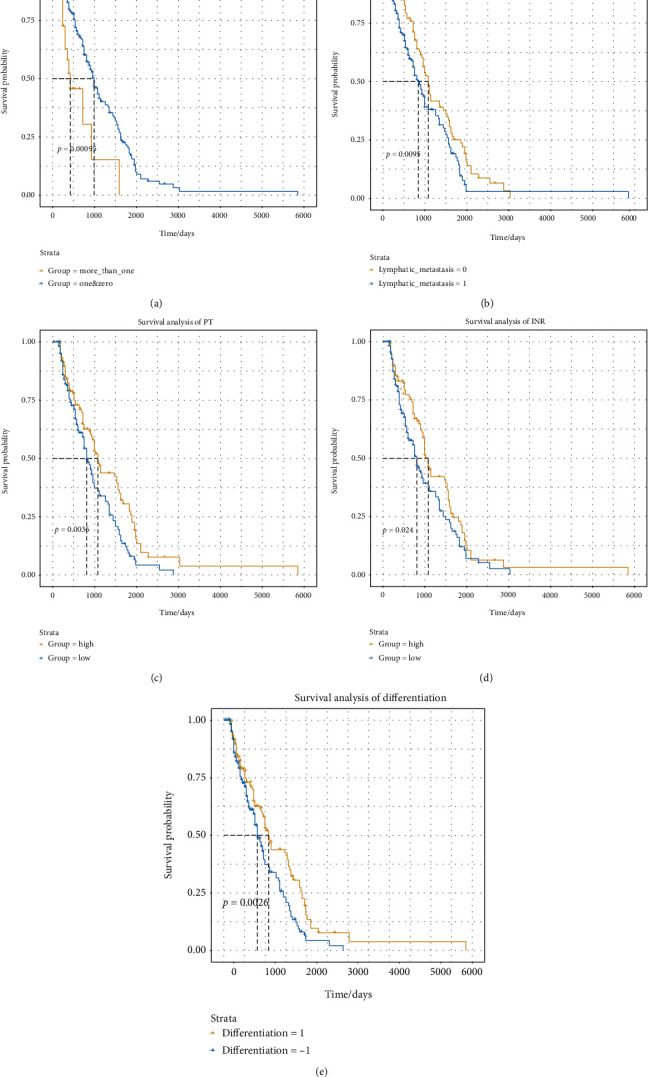
Kaplan-Meier survival curves related to (a) lymphatic metastasis, (b) INR, (C) PT, and (d) differentiation and (e) number of tumor metastases in the training set and validation set. INR: international standardized ratio; PT: prothrombin time.

**Figure 7:**
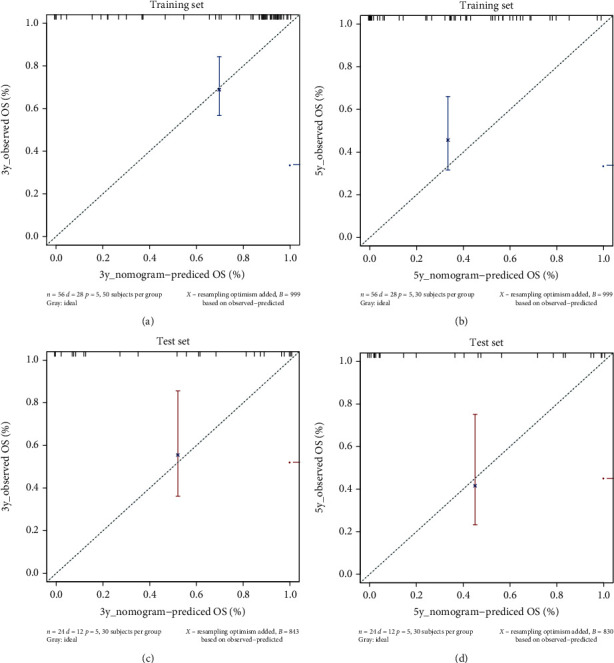
The calibration curves in predicting patient survival at (a) 3 years and (b) 5 years in the training set and at (c) 3 years and (d) 5 years in the test set. Nomogram-predicted probability of overall survival is plotted on the *x*-axis; actual overall survival is plotted on the *y*-axis.

The C-index results demonstrated that the model possessed a good ability in predicting the survival rate of NET patients in both the training set (C-index was 0.891) and test set (C-index is 0.804). In addition, the ROC results verified that the performance of the model was robust (training set: AUC value was 0.823. test set: AUC value was 0.768) (Figure 4). Additionally, as shown in Figure 5, the 3-year survival rate of 244 patients in the training set was 62.90% (95% CI: 56.7%-84.3%), while the 5-year survival rate was 29.7% (95% CI: 15.9%-55.4%). The patient's 3-year survival rate of 104 patients in the test set was 41.8% (95% CI: 31.2%-56.4%), while the 5-year survival rate was 27.8% (95% CI: 12.3%-62.9%). Afterwards, Kaplan-Meier survival curves were drawn for the five variables included in the nomogram: lymphatic metastasis, international normalized ratio, prothrombin time, tumor differentiation, and the number of tumor metastasis sites. The results showed that the five variables, lymphatic metastasis (*p* = 0.0095), international standardized ratio (*p* = 0.024), prothrombin time (*p* = 0.0036), tumor differentiation (*p* = 0.0026), and the number of tumor metastases (*p* = 0.00095), were all significantly correlated with survival status (Figure 6). Furthermore, the calibration curve results indicated that predictive values based on the nomogram (3-year survival rate and 5-year survival rate) and observed values in the training set and validation set exhibited strong consistency (Figure 7).

## 4. Discussion

In order to assess the biological behavior of NETs, the histopathological criteria used for diagnosis and classification have been widely established. However, due to heterogeneity of NETs, the lack of a recognized NET staging system has hindered the provision of prognostic information for patients. Currently, the most commonly used NET staging systems are the naming and classification standards developed by the WHO in 2010 and the TNM staging system developed by the AJCC. However, some limitations are present in these staging systems. For example, the current AJCC staging system only distinguishes 5-year OS between phase I and phase II (84% vs 72%. *p* = 0.01) and between phase 2 and phase 3 (72% vs 65%. *p* = 0.97). As a result, the third and fourth stages (65% vs 55%. *p* = 0.36) cannot be effectively distinguished [12]. In addition, these staging systems only focus on the survival rate of patients with NETs at specific sites. The purpose of the present research was to develop and preliminarily validate a nomogram for patients with NETs in order to accurately predict individual survival rates following treatment. Compared to traditional staging systems, a nomogram may serve as a more accurate and clinically viable tool in predicting cancer outcomes. Therefore, it is important to establish a nomogram model to predict the prognosis of all patients with NETs.

In this study, five variables (lymphatic metastasis, international normalized ratio (INR), prothrombin time (PT), tumor differentiation, and the number of tumor metastatic sites) were used to establish the nomogram model. Here, lymphatic metastasis was an independent prognostic factor related to patient survival; however, the impact of lymphatic metastasis on patient survival remains controversial. A multicenter study demonstrated that the presence or absence of lymphatic metastasis has nothing to do with liver metastasis or increased risk of survival [13]. In addition, a prospective single-institution study indicated that lymphatic metastasis had no significant effect on survival. Existing studies have considered the relationship between the number of lymphatic metastases and survival; however, only using two lymph nodes as the critical value is ambiguous and limited [14]. At the same time, numerous other studies have shown that a major feature of neuroendocrine tumors is its tendency to metastasize to the lymphatic system [15]. Lymphatic metastasis reduced the survival rate and is closely related to mortality; its prognostic significance is similar to that of the Ki67 proliferation index [16]. In this study, the HR of lymphatic metastasis was found to be 1.56 (95% CI, 1.03-2.38). In addition, compared to patients with lymphatic metastasis, the survival rate of patients without lymphatic metastasis was found to be significantly higher (*p* = 0.0095).

Patients with malignant solid tumors usually present with aberrant activation of the blood coagulation system, resulting in an elevated incidence of thromboembolism [17]. PT and INR are two blood coagulation markers that can be used to predict the outcomes of certain cancers [18]. In this study, a multivariate analysis demonstrated that the HR of PT was 0.59 (95% CI: 0.4207-0.8755), indicating that for every percentage increase in PT, the risk of death reduced by 41%. In addition, the survival rate of patients with a longer PT was found to be significantly higher than that of patients with a shorter PT (*p* = 0.0036). The international normalized ratio (INR) refers to the ratio of prothrombin time of a patient to the normal control prothrombin time to the ISI power (ISI: international sensitivity index). When the same sample is tested in different laboratories with different ISI reagents, the PT value results are usually very different. However, the measured INR values are the same, increasing the comparability of the measured results [19]. In patients with malignant tumors, the coagulation index also changed; hence, it is possible to use INR to detect the prognosis of patients with NETs.

The heterogeneity of tumors is divided according to the degree of tumor deterioration compared to normal tissue morphology. The WHO 2010 classification defined well-differentiated neuroendocrine and poorly differentiated tumor based on the Ki67 proliferation index and mitotic index [20]. A NET is defined as a well-differentiated neuroendocrine neoplasm resembling normal gut–pancreas endocrine cells [21]. In contrast, a neuroendocrine carcinoma (NEC) is defined as a poorly differentiated, high-grade malignant neoplasm composed of small or large to intermediate cells [22]. In addition, the type of pathology (high, medium, and low differentiation) is recommended by various guidelines in predicting the prognosis of neuroendocrine tumor patients [23]. Therefore, the degree of tumor differentiation can be used as a variable in order to predict the prognosis of neuroendocrine tumor patients.

Among NETs, metastatic cancer refers to the spread of neuroendocrine tumors from the primary site to another part of the body, in which liver metastasis is more common. Studies have shown that liver metastasis is one of the predictors of poor prognosis in patients with NETs [24]. Up to about 20% of patients have metastasis at the tumor site with a significant difference in metastatic potential. Most patients with pancreatic and small intestinal neuroendocrine tumors are diagnosed with metastatic status [25]. Here, metastasis was observed to play an important role in the development of neuroendocrine tumors. In addition, the number of tumor metastatic sites was found to be closely related to the prognosis of patients. The OS rate of patients with two or less metastatic sites was observed to be significantly better than that of one tumor site (*p* = 0.00095).

Evidently, the ROC results of this prediction model demonstrated that the prediction performance of the model was sufficiently robust in the training set (AUC value = 0.823) and test set (AUC value = 0.768). Moreover, the C-index results illustrate that the model constructed in this study possessed a certain robustness. In addition, in other nomogram models that predict the survival rate of patients with NETs at specific sites, the prognostic risk factors were mostly the Ki67 proliferation index and CgA [26]. Additionally, in this study, the variables used to build a prediction model were lymphatic metastasis, INR, PT, tumor differentiation, and the number of tumor metastasis sites. As a result, this study may provide new insights into the prediction of the OS rate of NET patients.

Some limitations are present in this study; however, first, this investigation may be affected by limitations associated with all retrospective studies and single-center studies. However, as a relatively rare tumor, the number of cases studied was relatively large, which is of great significance. Second, although a nomogram was developed and validated that can predict the survival rate of NET patients, its reliability needs to be verified in a larger cohort. In addition, the risk factor analysis did not include all potential factors affecting the survival of patients suffering from NETs. At the same time, the collected cases were not large enough, and a larger cohort of data should be collected in order to optimize the performance of the model.

## 5. Conclusion

Based on five variables (lymphatic metastasis, international standardized ratio, prothrombin time, tumor differentiation, and the number of tumor metastatic sites), the proposed nomogram was shown to predict the 3-year and 5-year survival rates of patients with NETs. In addition, the above five variables were significantly found to be correlated with survival rate. The C-index (training set: 0.890, test set: 0.804), ROC curve analysis results (training set: 0.828, test set: 0.768), and calibration curve all verified the robustness of the model. Therefore, the proposed model may be clinically helpful in predicting the prognosis of patients suffering from NETs.

## Figures and Tables

**Table 1:** Baseline characteristics (*N* = 348).

Patients	All cohort	Training cohort	Test cohort	*p* value
All patients	348	244	104	-
Age				
<56	172	120	52	>0.05
≥56	176	123	53	
Gender				
Male	196	137	59	>0.05
Female	152	106	46	
Tumor primary site				
Lung	106	74	32	>0.05
Gastrointestinal tract	61	43	18	
Pancreas	14	10	4	
Cervix	57	40	17	
Other	138	97	41	
Tumor metastasis site				
No	259	181	78	>0.05
Yes	89	62	27	
Liver metastasis	38	27	11	
Lung metastasis	13	9	4	
Other	38	27	11	
Number of tumor metastasis sites				
≤1	232	162	70	>0.05
>1	116	81	35	
Chemotherapy				
Yes	245	172	74	>0.05
No	103	72	31	
Differentiation				
High	66	46	20	>0.05
Low	282	197	85	
Grade				
G1-2	68	48	20	>0.05
G3	280	196	84	
Lymphatic metastasis				
No	108	76	32	>0.05
Yes	240	168	72	
Nerve invasion				
No	308	216	92	>0.05
Yes	40	28	12	
HBSAg				
No	284	199	85	>0.05
Yes	64	45	19	
HCV				
No	346	242	104	>0.05
yes	2	1	1	
HBeAg				
No	347	243	104	>0.05
yes	1	1	0	
HBeAb				
No	337	236	101	>0.05
yes	11	8	3	
HBcAb				
No	331	232	99	>0.05
yes	17	12	5	
AST	0.7~126	10~126	0.7~118	>0.05
ALP	15~304	24~338	15~322	>0.05
BUN	1.2~16.68	1.2~16.68	1.4~33.2	>0.05
Creatinine	30~163	35~202	10~163	>0.05
CEA	0.36~66.26	0.29~66.26	0.2~98.79	>0.05
AFP	0.93~27	1.03~84.96	0.93~97.43	>0.05
CA125	2.6~97.7	2.6~94.7	2~78.2	>0.05
CA153	1.3~79	1.3~79	4.2~76.8	>0.05
CA199	0.2~93.87	0.1~120	0/2~117.6	>0.05
Ki67 proliferation index	0.1~0.99	0.01~0.99	0.08~0.98	>0.05

**Table 2:** Univariate analysis, 3-year survival rate, and 5-year survival rate.

Variable	*p* value	HR (95% CI)	3-year survival rate (95% CI)	3-year survival rate (95% CI)
Grade	0.0063	0.65 (0.47-0.88)		
G3 (reference)			37.08% (26.86%-51.2%)	5.06% (1.68%-15.2%)
G1-2			44.1% (36.7%-53.1%)	27.8% (20.6%-37.6%)
Lymphatic	0.0096	1.5 (1.1-2.1)		
No (reference)			48.4% (38.8%-60.5%)	25.1% (17%-37%)
Yes			38% (30.11%-47.9%)	14.3% (8.33%-24.7%)
Differentiation	0.003	1.3 (1.1−1.5)		
High (reference)			45.2% (37.8%-54.1%)	28.1% (20.9%-37.9%)
Low			3.94% (24.783%-49.3%)	3.49% (0.898%-13.7%)
CEA (ng/mL)	0.00012	1 (1-1)		
Normal (<5.0)			38.4% (29.3%-50.4%)	20.1% (12.3%-33%)
Abnormal			44.5% (36.4%-54.5%)	19% (12.6%-28.5%)
Hemoglobin (g/L) (male)	0.016	1 (1-1)		
Normal (120-165)			25.24% (16.5%-38.6%)	8.41% (3.4%-20.8%)
Abnormal			62.4% (48.6%-80%)	38.6% (23.6%-63.2%)
Hemoglobin (g/L) (Female)	0.016	1 (1-1)		
Normal (110-150)			39.7% (28.7%-54.8%)	16.7% (9%-31%)
Abnormal			56% (43.3%-72.5%)	24.7% (13.5%-45.3%)
Creatinine (*μ*mol/L)	0.012	0.99 (0.98-1)		
Male				
Normal (60–110)			0	0
Abnormal			40.3% (23.8%-68.4%)	26.9% (10.3%-70.1%)
Female				
Normal (45-90)			0	0
Abnormal			53.4 % (34.9%-81.8%)	33.9% (17.3%-66.6%)
PT(s)	0.00037	0.81 (0.73-0.91)		
Normal (12–14)			47.5% (36.9%-61.1)	33% (22.8%-47.8%)
Abnormal			38.9% (31.45%-48%)	12.4% (7.48%-20.7%
INR	0.0048	0.2 (0.065-0.61)		
Normal(0.8 - 1.2)			40.8% (34.2%-48.7%)	18.9% (13.5%-26.4%)
Abnormal			49.4 % (32.61%-74.9%)	21.6% (8.46%-55.3%)
Total bilirubin (*μ*mol/L)	0.00055	1 (1-1)		
Normal (3.4-17.1)			42%(35.4%-50%)	19.3%(13.8%-27%)
Abnormal			38.9% (23.79%-63.7%)	19.5% (8.32%-45.6%)
AST (U/L)	0.045	1 (1-1)		
Normal (8-40)			41% (34.2%-49.1%)	17.8%(12.5%-25.5%)
Abnormal			45.4% (31.2%-66%)	26.5% (13.7%-51.1%)
Tumor metastasis	0.023	1.2 (1−1.4)		
No			52.91% (44.5%-59.1%)	42.36% (30.2%-48.1%)
Yes			40.98% (36.8%-48.2%)	38.25% (32.2%-45.5%)
Number of tumor metastases	0.00022	1.5 (1.2−1.8)		
≤1			43.4% (36.9%-51.1%)	20.3% (14.8%-27.8%)
>1			0	0

**Table 3:** Multivariate analysis and independent predictors related to survival.

Variable	HR	*p* value	Lower .95 CI	Upper .95 CI
Tumor metastasis site	1.35	0.5241	1.19	2.42
Number of tumor metastases	4.15	0.01676	1.29	13.34
Grade	1.12	0.313936	0.25	0.72
Lymphatic metastasis	6.57	0.000324	5.71	16.94
CEA	1.01	0.202226	1.00	1.01
Hemoglobin	1.02	0.055991	1.00	1.04
PT	0.05	0.00182	0.01	0.32
INR	1.93	0.005133	1.81	1.95
Total bilirubin	0.88	0.189559	0.74	1.06
Differentiation	3.76	0.011088	1.68	15.67

**Table 4:** Multivariate analysis and independent predictors related to survival.

Variable	HR	*p*	Lower .95 CI	Upper .95 CI
Number of tumor metastases	4.15	0.01676	1.29	13.34
Lymphatic metastasis	6.57	0.000324	5.71	16.94
PT	0.05	0.00182	0.01	0.32
INR	1.93	0.005133	1.81	1.95
Differentiation	3.76	0.011088	1.68	15.67

## Data Availability

The raw data used to support the findings of this study are available upon author's request.
